# The STROBE guidelines and the Αristotelian mesotes curve

**DOI:** 10.1007/s42000-026-00758-3

**Published:** 2026-05-04

**Authors:** George P. Chrousos, Nektaria Papadopoulou-Marketou, Panagiotis Tsiamyrtzis

**Affiliations:** 1 University Research Institute of Maternal and Child Health and Precision Medicine, UNESCO Chair on Adolescent Health Care, National and Kapodistrian University of Athens, and Hellenic Pasteur Institute, Athens, Greece; 2 Neuroendocrine Tumor Unit, Department of Propaedeutic and Internal Medicine, Laiko General Hospital, ENETS Centre of Excellence, National and Kapodistrian University of Athens, Athens, 1st, 11527 Greece; 3 University Research Institute of Maternal and Child Health and Precision Medicine, National and Kapodistrian University of Athens, Athens, Greece; 4 Department of Mechanical Engineering, Politecnico di Milano, Milan, Italy; 5 Department of Statistics, Athens University of Economics and Business, Athens, Greece

**Keywords:** Aristotle, Bayesian analyses, Bonferroni correction, False discovery rate (FDR), Statistical guidelines, STROBE guidelines

## Abstract

The STROBE (Strengthening the Reporting of Observational Studies in Epidemiology) guidelines aim to improve the reporting of observational studies, including cohort, case-control, and cross-sectional designs, relevant to assessing associations, risks, and outcomes in real-world clinical settings. They are conceived to optimize the meaningfulness of epidemiological and clinical studies, aligning them with the Aristotelian “mesotes” curve, namely, the principle of achieving balance and avoiding extremes to best reflect *the most truthful* approximation of reality. This commentary addresses situations where strict adherence to STROBE guidelines may be impossible or inappropriate, potentially distorting results, and shows how statistical tools can mitigate these issues. Specific challenges arise in real-life randomization scenarios, situations lacking placebo arms, and in correcting for multiple comparisons. We discuss these challenges, examine the role of Bonferroni and similar corrections, and propose alternative approaches such as false discovery rate (FDR) and Bayesian hierarchical models. We illustrate these points with examples from the literature and a simulation study evaluating p-value adjustments in multiple hypothesis testing. This work provides a framework for researchers to navigate STROBE guidelines thoughtfully, ensuring that observational studies are both rigorous and relevant.

## Introduction

The purpose of the STROBE guidelines is to ensure that observational studies are reported completely, transparently, and consistently so that readers can assess the study’s validity, bias, and applicability – in other words, its relevance to the optimal, the ideal, the most truthful approximation to reality. The STROBE Checklist – Key Components (22 items in total) is organized into the standard sections of a research article, as follows: Title and Abstract clearly state the study design; the Introduction provides the background and rationale and the objectives and hypotheses; Methods provide the study design, setting, participants, variables, data sources, and measurements, as well as the possible bias, study size, and statistical methods; Results contain the participant flow, descriptive data, outcome data and main results (including confounder adjustment), and sensitivity analyses; Discussion provides the key results, limitations, interpretation, and generalizability of the findings; Funding and conflicts of interest are totally transparent. However, strict adherence to STROBE guidelines may not always be feasible or optimal. This commentary explores situations where a more nuanced approach to STROBE, coupled with appropriate statistical considerations, may be necessary to accurately represent study findings. We frame this discussion within the concept of the Aristotelian “mesotes” curve. The mesotes curve, or the principle of the golden mean, suggests that the optimal point lies in the balance between two extremes. In the context of observational studies, this means that blindly following every STROBE recommendation may sometimes lead to a misrepresentation of the data, just as completely ignoring the guidelines would.

Several factors can hinder full compliance with all STROBE prerequisites. These include the following. (1) Lack of awareness or training, as many researchers, especially early-career investigators or those outside epidemiology, may not be familiar with STROBE; Inadequate training in research methodology or scientific writing can lead to poor reporting quality. (2) Journal limitations, as word limits in journals can restrict detailed descriptions, while some journals do not enforce STROBE or lack a strong editorial policy supporting reporting guidelines. (3) Time and resource constraints, as researchers may rush the writing process due to funding deadlines or publication pressure or have limited access to statisticians or methodologists. (4) Study design or data limitations given that retrospective studies or secondary data analysis may lack certain key variables, while incomplete or poor-quality data may prevent full adherence to items like confounder control or detailed follow-up information. (5) Poor documentation or planning, as studies not initially designed with STROBE in mind might miss important aspects needed for proper reporting (e.g., justification for sample size, handling of missing data), while missing protocols or lack of a pre-registered analysis plan can make it hard to follow STROBE standards. (6) Perceived redundancy or misinterpretation, as authors may consider some checklist items redundant or assume they are self-evident, while misunderstanding of STROBE items can lead to partial or incorrect reporting. (7) Editorial or peer review oversight; if editors and reviewers do not enforce adherence to STROBE, authors may not be held accountable for complete reporting.

Examples of STROBE items that are commonly underreported or misreported include the following. (1) Studies often fail to clearly describe eligibility criteria, sources, and methods of participant selection. Lack of such transparency can obscure selection bias or generalizability concerns. (2) Many studies do not adequately describe efforts to address potential sources of bias (e.g., selection bias, information bias), understanding bias being crucial for assessing the validity of findings. (3) The statistical methods employed in the evaluation of the data is key, and incomplete reporting of how confounding was addressed or how missing data were handled cannot be emphasized. Without proper statistics, readers cannot assess the robustness of the analyses. (4) Authors often report unadjusted estimates without presenting confounder-adjusted results or failing to clearly specify which confounders were adjusted for. This makes interpretation and comparison across studies difficult. (5) Some studies either downplay limitations or fail to discuss key weaknesses, such as potential biases, measurement errors, or limited generalizability. Full disclosure of limitations allows for a more accurate appraisal of the evidence. (6) There is frequent under-reporting or vague reporting of funding sources and of the role of funders, which limits transparency and hampers the assessment of potential conflicts of interest. Transparency around funding helps assess potential conflicts of interest.

In this commentary, we explore specific scenarios where strict adherence to STROBE guidelines might be counterproductive. These include situations involving challenges in randomization, the use of placebo arms, and corrections for multiple comparisons. In each case, we discuss how researchers can navigate these challenges by carefully balancing STROBE recommendations with appropriate statistical methods with the aim of achieving the mesotes – the optimal balance for valid and meaningful research. A concise overview of the main contexts in which strict STROBE adherence may be impractical, along with appropriate methodological alternatives, is summarized in Table 1.

**Table 1:** Situations where strict STROBE adherence may be challenging and alternative approaches

Context	Example of limitation	Reasons that STROBE criteria are difficult to apply	Alternative design / statistical approach
Randomization	Public health interventions (e.g., fluoridation)	Random assignment at population level is infeasible	Natural or quasi-experimental design; Difference-in-Differences
	Rare diseases (e.g., progeria)	Very small patient populations	Historical controls; Single-arm trial
	Life-saving or emergency treatments	Randomizing to no-treatment is unethical	Cluster or stepped-wedge randomization
	Educational / behavioral interventions	Institutional refusal or logistic constraints	Stepped-wedge or matched cohort design
	Surgical techniques / devices	Surgeon or patient preference	Registry or propensity-score matched studies
	Observational cohorts (e.g., smoking in pregnancy)	Randomization unethical	Cohort analysis with confounder adjustment
Placebo arm use	Surgical procedures	Sham surgery unethical	Active comparator or non-placebo control
	Emergency treatments	Withholding effective therapy unethical	Historical or active comparator
	Palliative/end-of-life care	Placebo in distressing symptoms inhumane	Observational comparative effectiveness study
	Invasive neurologic/psychiatric procedures	Blinding impossible; sham unethical	Crossover or delayed-start design
	High-risk infections (e.g., Ebola vaccine)	Placebo unethical during outbreak	Ring vaccination; active comparator
Multiple comparisons	Multiple outcomes, biomarkers, subgroups	Inflation of false positives	Adjustments via Bonferroni, Holm, FDR, Empirical Bayes; interpret contextually
Balance concept (mesotes)	—	Excessive strictness may suppress meaningful findings; too little rigor increases false positives	Adopt balanced use of corrections aligned with study goal

## The Aristotelian mesotes curve

**Fig. 1:**
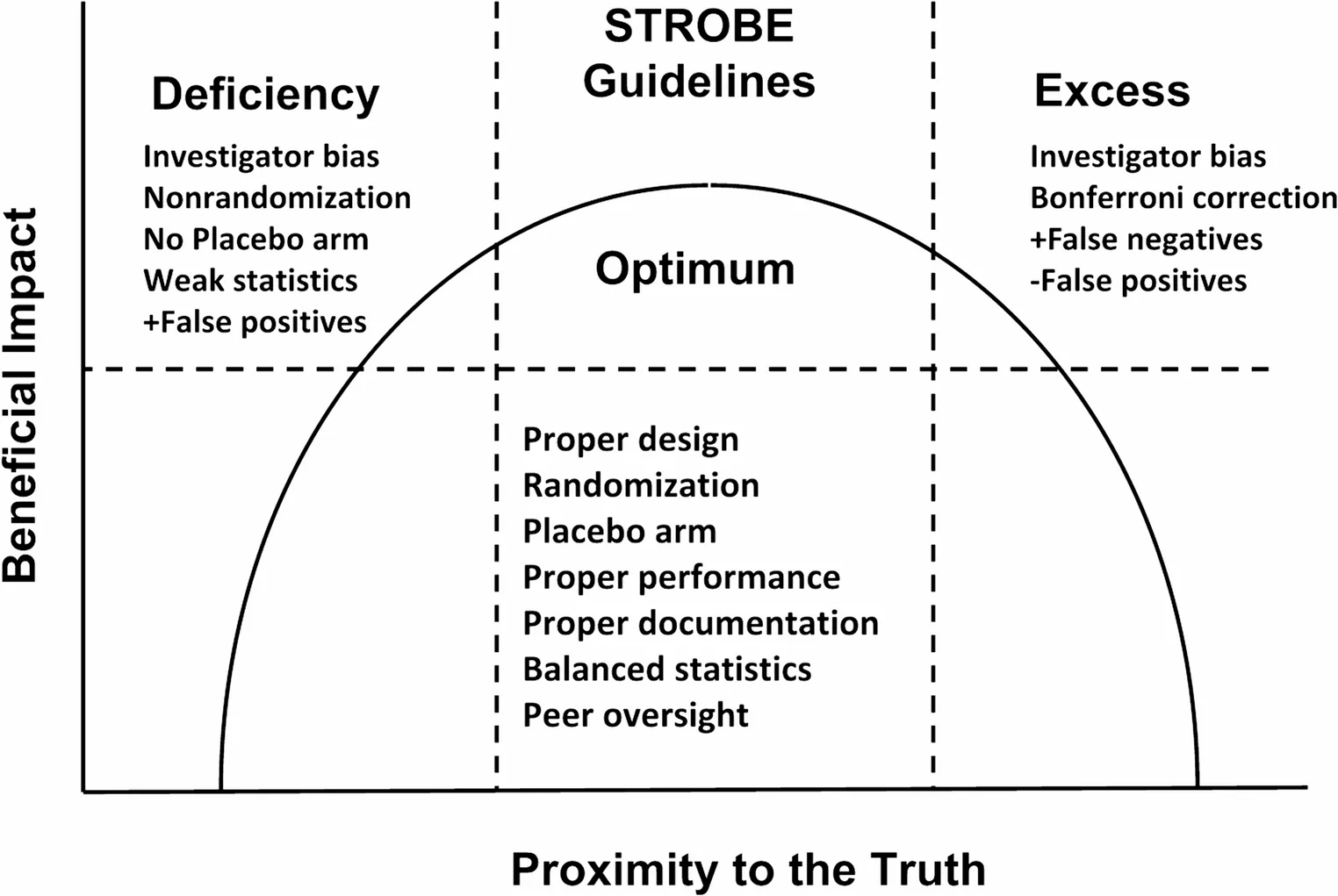
The Aristotelian mesotes curve, with “the good lying between two evils”, respectively, deficiency (too little) and excess (too much), both states that lie at a distance from the most truthful approximation to reality and, hence, are of low benefit to human society. STROBE strives to provide maximum benefit from a study by keeping its conclusions closest to the ideal, most truthful approximation to reality.

The STROBE guidelines represent an attempt to optimize the meaningfulness of epidemiological and clinical studies – i.e., their ability to reflect the optimal, the ideal, the most truthful approximation of reality – by applying the Aristotelian concept of mesotes, or the golden mean. The ultimate aim of the scientific process in its search for the best approximation of the optimal is to maximize its benefit to society (Fig. 1). However, real-life situations can create scenarios where strict, uncritical adherence to STROBE guidelines may inadvertently lead to a diversion from this optimal balance. We discuss three such situations where appropriate statistical handling of the data can help mitigate these deviations, namely, challenges in randomization, limitations in the use of placebo arms, and the need for corrections for multiple comparisons. We place particular emphasis on the challenges of multiple comparisons and the application of the Bonferroni or similar corrections. While acknowledging the importance of the Bonferroni adjustment, we recognize that its overly strict application could potentially nullify the conclusions of many previously published studies, including studies that have positively influenced current medical practice. Therefore, a balanced perspective is essential to avoid unintended negative consequences.

### Randomization in clinical studies

Randomization is a cornerstone of rigorous clinical research. However, ethical, logistical, or practical reasons can make randomization difficult or impossible in many real-world situations. In these cases, strict adherence to STROBE guidelines, which emphasize the importance of randomization, may not be feasible. Researchers must then rely on alternative study designs and statistical methods to minimize bias and approximate causal inference. The following examples illustrate situations where randomization is challenging and highlight alternative approaches that can be used to generate meaningful evidence.

1. Public health interventions: Consider the example of fluoridation of community water supplies. Randomly assigning entire cities to receive or not receive fluoridated water is often impossible due to logistical constraints, infrastructure changes, and the need for community consent. In such cases, researchers often employ natural experiments or quasi-experimental designs, comparing communities with and without fluoridation. Numerous epidemiological studies have examined dental caries rates in fluoridated versus non-fluoridated regions [1].
2. Rare diseases or conditions: Studies of rare diseases, such as Batten disease or Hutchinson-Gilford Progeria, often face challenges with randomization due to the limited number of affected patients globally. Random assignment may not be feasible while still achieving adequate statistical power. In these situations, researchers may turn to historical controls or single-arm trials where all patients receive the treatment. The Lonafarnib trials for Progeria [HGPS] [2], which used historical controls due to the ultra-rare nature of the condition, provides an example of this approach.
3. Life-saving treatments (ethical constraints): For ethical reasons, randomization is often impossible when evaluating life-saving treatments. For example, in the case of CPR (cardiopulmonary resuscitation) techniques in cardiac arrest, it would be unethical to randomize patients to “receive” versus “not receive” CPR in an emergency. In such cases, researchers may use cluster-randomized trials (e.g., by EMS unit) or before-and-after studies. The ROC PRIMED trial [3], which used cluster randomization of EMS units to different airway management strategies, provides a useful example.
4. Educational or behavioral interventions: Randomization can be challenging when evaluating educational or behavioral interventions, such as smoking cessation campaigns in schools. Schools or communities may refuse to be randomized or have policies against assigning interventions arbitrarily. Stepped-wedge designs or matched cohort studies may provide viable alternatives. Several tobacco prevention studies [4]. have used quasi-experimental designs to compensate for this problem.
5. Surgical techniques or device trials: When evaluating new surgical techniques or devices, such as new surgical robots or implants, randomization can be difficult. Surgeons may have strong preferences or expertise biases, and patients often request the “new” option. Registry-based studies or propensity-score matched cohorts can provide valuable data in these situations. TAVI (transcatheter aortic valve implantation) registries provided reliable results even before RCTs were conducted [5, 6].
6. Observational studies with no ethical alternative: In some cases, ethical considerations may preclude randomization altogether. For example, it is impossible to randomize pregnant women to smoke or not smoke to study the impact of maternal smoking on fetal development. Researchers must rely on observational cohorts with statistical controls for confounders. Multiple birth cohort studies, like ALSPAC [7, 8] (UK) or the Danish National Birth Cohort [9, 10], provide examples of this approach and have yielded credible results.

Statistical methods to compensate for lack of randomization

When randomization is not possible, researchers rely on a variety of statistical methods and study designs to minimize bias and approximate causal inference. Key methods include the following.

1. Propensity score methods: These methods adjust for confounding by balancing observed covariates between treatment groups. They estimate the probability of receiving treatment given observed covariates and then use that score to adjust. Techniques include the following: 1. Matching (e.g., matching each subject treated to one or more controls with similar propensity scores). 2. Stratification (grouping subjects into strata by score). 3. Weighting (inverse probability of treatment weighting). 4. Covariate adjustment (including the score as a covariate in regression). They are used in comparative effectiveness research, registry studies, and retrospective analyses.
2. Regression adjustment (multivariable modeling): These methods adjust for differences in baseline characteristics using multiple regression (e.g., linear, logistic, Cox). The pros are flexibility, broader understanding, and deeper insight; the cons are that they only adjust for measured confounders and results depend on model specification. They are used in observational cohort studies and EHR-based analyses.
3. Instrumental variable (IV) analysis: This approach uses a variable (the “instrument”) that affects treatment choice but is unrelated to the outcome except through the treatment. An example is the distance to a hospital offering a certain treatment as an instrument. The pros are that it can address unmeasured confounding; the cons are that valid instruments are rare and hard to find. They are used in health economics and pharmacoepidemiology.
4. Difference-in-differences (DiD): This method compares changes in outcomes over time between treated and untreated groups under the assumption that they would have followed parallel trends without intervention. It is used in policy evaluations and natural experiments. An example is assessing the effect of Medicaid expansion on health outcomes across the states of the USA .
5. Interrupted time series (ITS): This approach analyzes trends before and after an intervention in a single group or system. It is powerful when there are a clear intervention point and enough data points pre/post and is used in public health policy evaluation (e.g., seat belt laws, vaccination mandates).
6. Matching methods (Beyond Bayesian propensity scores): Matching methods (Bayesian and related propensity-score approaches): Exactly matching pairs of subjects who share identical values for specified covariates, ensuring perfect balance on these variables.. Mahalanobis distance matching (MDM) uses a multivariate distance metric for similarity and coarsened exact matching (CEM) temporarily groupsvariables into bins to improve matching balance.
7. Synthetic control methods: This approach builds a weighted combination of control units to create a “synthetic” control that mimics the treatment group before intervention and is used in evaluating interventions at the population or policy level (e.g., effect of a new drug policy in one country).
8. Sensitivity analyses for unmeasured confounding: This approach evaluates the robustness of findings as compared to hypothetical levels of unmeasured confounding and is used in high-stakes observational research to assess the credibility of causal claims.
9. Target trial emulation: This comprises an observational study designed to mimic a hypothetical randomized trial as closely as possible. The framework includes clear eligibility criteria, treatment strategies, follow-up period, and analysis plan and is used in EHR studies and real-world data applications (e.g., emulating cardiovascular outcome trials using claims data).

## The use of placebo arms in clinical research: ethical and practical limitations

While placebo-controlled trials are considered a gold standard in clinical research, there are many situations where the use of a placebo arm is impossible or unethical. In these cases, strict adherence to STROBE guidelines that promote placebo controls may be inappropriate. Researchers must instead consider alternative study designs and comparators to rigorously evaluate interventions. The following examples illustrate these challenges.

Surgical interventions: In surgical trials, such as those evaluating coronary artery bypass grafting (CABG), it is generally not ethically or practically feasible to perform sham open-heart surgery as a control. Instead, these trials typically compare one surgical method to another (e.g., on-pump vs. off-pump CABG). The ROOBY Trial [11], for example, compared on-pump vs. off-pump surgery without a placebo arm.

Emergency treatments: Withholding potentially life-saving emergency treatments, such as thrombolysis for acute ischemic stroke, where administration of a placebo would be unethical, particularly when a therapy has already demonstrated effectiveness. Historical controls or active comparator trials may be used instead. The NINDS tPA Stroke Trial [12] initially used a placebo arm, but subsequent studies deemed this unethical once the benefit of tPA was established.

Palliative and end-of-life care: Using a placebo instead of active sedatives in palliative and end-of-life care for terminal restlessness or pain is considered inhumane. Observational studies or comparative effectiveness studies of different sedation regimens are more appropriate in this context. Many palliative care trials are non-randomized due to ethical considerations, for example, studies comparing midazolam to other agents [13].

Certain psychiatric or neurologic interventions: For invasive psychiatric or neurologic interventions, such as deep brain stimulation (DBS) for Parkinson’s disease, blinding is difficult and sham surgery raises ethical concerns. Crossover designs or delayed-start designs are often used instead. Deuschl et al. [14] studied DBS vs. best medical therapy rather than placebo.

Vaccine efficacy in high-risk settings: In high-risk settings, such as Ebola vaccine trials during outbreaks, it is often unethical to give people at high risk of a deadly disease a placebo. Ring vaccination designs or the use of active comparators may be more appropriate. The “Ebola ça suffit!” trial [15] used ring vaccination, not a placebo, due to urgency and high mortality rates.

## Bonferroni and similar corrections for multiple comparisons in clinical research

In clinical studies, multiple hypothesis tests are often performed (e.g., multiple endpoints, subgroup analyses, or biomarker comparisons). The Bonferroni correction is a common method for adjusting for multiple comparisons by using a more stringent significance threshold (α/n), where α denotes the nominal significance level and n is the number of tests. This approach aims to control the family-wise error rate (the probability of at least one false positive) at a desired level, typically 0.05. However, the trade-off is that requiring smaller p-values may cause previously significant results to become non-significant. In practice, the conservativeness of the Bonferroni correction is well recognized: it reduces false positives but increases the risk of false negatives. Numerous real-world clinical studies across oncology, cardiology, and psychiatry have demonstrated that applying Bonferroni adjustments can substantially alter study outcomes [16].

## Implications for clinical decision-making: navigating the Bonferroni correction and beyond

The examples below underscore the important implications of using strict Bonferroni correction in clinical research, but also highlight the need for a broader perspective on multiple comparisons.

Stricter thresholds and lost significance: By dividing α by the number of comparisons, Bonferroni raises the bar for significance. Results that would be considered significant at *p* < 0.05 may be rendered non-significant if they do not exceed the new threshold (e.g., 0.005 or 0.001, depending on multiplicity). In practice, nominally positive findings can disappear after correction. This can markedly change study conclusions: for instance, an association or treatment effect that researchers might have otherwise touted must be downplayed or rejected when it does not survive the correction. Clinicians relying solely on corrected results might never hear about those borderline findings.

The benefit of robustness: The clear benefit of Bonferroni adjustment (and similar corrections) is robust control of Type I error. Any result that remains significant after Bonferroni adjustment is very unlikely to be a false positive. This increases confidence in positive findings. In clinical decision-making, this means that if a finding is reported as significant with Bonferroni in place, one can be more confident that it is not just a fluke due to multiple testing. This conservatism is especially valued in confirmatory phase III trials or meta-analyses where acting on a false positive (e.g., adopting an ineffective therapy) would have serious consequences.

The risk of over-correction: The flip side is that Bonferroni is very conservative, often considered an overly harsh penalty when many tests are conducted. It does not account for any relationship between outcomes (assuming all tests are independent) and, thus, can be too strict if outcomes are correlated. This results in a higher risk of Type II errors, with real effects being missed. As Perneger noted, the likelihood of Type II errors is increased, so that truly important differences are deemed non-significant. In our examples, potentially meaningful associations (genetic toxicity predictors or secondary benefits of therapy) may have been overlooked due to a lack of power to meet the corrected α. For clinical evidence, this implies that some findings labeled “non-significant after correction” might be genuine effects in an underpowered study. If researchers or clinicians interpret non-significance strictly, they might dismiss a real effect (e.g., ignoring a promising biomarker or discontinuing the development of a therapy that actually works for a subset). Thus, Bonferroni can shift the balance toward false negatives, which is a notable concern in fields where the discovery of new signals is important (e.g., early-phase exploratory studies). The simulation study that follows further illustrates this point.

Conservative conclusions and need for replication: Applying a Bonferroni correction often forces a more cautious interpretation of data. Findings that do not meet the adjusted threshold are typically reported as exploratory or hypothesis-generating rather than definitive. For instance, the lung cancer biomarker study above had to suggest that subgroup findings “warrant further investigation” instead of claiming a discovery because most signals did not survive correction. In practice, this means that clinical decisions are less likely to change based on a single study with uncorrected *p* < 0.05 if that study appropriately corrects for multiplicity: only the clearest signals would drive changes in guidelines or practice. This places the onus on follow-up studies or larger trials to confirm any effects that were borderline. While this conservatism guards against false leads, it may slow the uptake of potentially effective interventions until more evidence accumulates.

Context matters: alternative approaches to multiple comparisons: The impact of Bonferroni also highlights why its use should align with the study’s purpose. In confirmatory trials (regulatory decisions, pivotal phase III), a strict approach (Bonferroni or other family-wise error control) is usually justified to avoid false claims. However, in more exploratory research (e.g., screening many biomarkers, early-phase studies), many experts caution against over-adjustment as it could hide interesting signals that merit follow-up. Some statisticians argue that it is often better to report all p values with transparency about the number of tests rather than automatically discard findings via Bonferroni, especially when the tests are not independent. Alternative methods like the false discovery rate (FDR) control are sometimes used in order to be less stringent than Bonferroni while still limiting false positives. Hierarchical testing strategies can also prioritize certain outcomes for formal testing and treat others as exploratory. In exploratory observational analyses, FDR or empirical Bayes methods may better approximate the mesotes balance than Bonferroni. Examples of other techniques that could be considered are the Holm-Bonferroni and Bayesian hierarchical model corrections. The advantages and the disadvantages of each method should be carefully assessed before being used.

The key for clinical decision-making is understanding whether a study did adjust for multiplicity and how that influences which results are considered “real.” If a published trial reports an outcome as significant and we know a strict correction was applied, we can have greater confidence in it; conversely, if an outcome just barely missed significance due to Bonferroni, one might consider looking at the effect size and confidence interval to judge clinical relevance rather than dismissing it outright.

The Bonferroni correction plays a critical role in how we interpret evidence across medical research domains, as illustrated in the following.

Real-world impact on results: In oncology, cardiology, psychiatry, and beyond, Bonferroni adjustments have turned seemingly significant results into non-significant ones. Examples include genetic associations with chemotherapy side effects which, in a trial, lost significance after correction and therapy benefits that were no longer significant once multiple outcomes were accounted for. Such cases demonstrate that study outcomes and conclusions can change dramatically depending on whether and how a multiple comparison correction is used.

Ensuring robust findings: The strength of the Bonferroni approach is its rigorous control of false positives. Any finding that remains significant after a Bonferroni correction is likely to be a true effect, which boosts confidence among clinicians. This helps prevent the premature adoption of interventions or biomarkers based on fluke results. Only the most pronounced effects (with very low p-values) survive, for instance, key outcomes in the psychiatric trial that stayed significant.

Trade-off: missing true effects: A strict Bonferroni correction can “wash out” real but modest effects. By raising the threshold for significance, studies risk failing to detect some true positives, potentially delaying or missing beneficial discoveries. Researchers and decision-makers must be aware that “non-significant” does not always equal “no effect,” especially if a study tested many hypotheses.

Implications for evidence interpretation: When evaluating clinical evidence, consider whether results were adjusted for multiple comparisons. If a trial’s secondary endpoint is reported as not significant, was it because there was truly no effect, or because a very strict correction was applied? For example, guideline committees might give less weight to a secondary analysis that fails Bonferroni significance, even if it showed a trend. On the other hand, something that meets Bonferroni-corrected significance provides stronger evidence to act on.

Real-world example: the authors’ study published in the Journal of Clinical Endocrinology and Metabolism [17]: After productive debate with the Journal Editorial Board, the decision was made to present the impact of brief, low-impact, high-intensity osteogenic loading (“Osteostrong^R^’’) on the density and quality of the bones of women with postmenopausal osteoporosis, after Bonferroni analysis. Thus, we took a reserved approach, emphasizing the most positive effects, and suggested further studies for duplication of the findings and recovery of positiveness possibly lost by the overstrict analysis. In this case, we will more closely approach the optimum of the Aristotelian curve! Notably, the three major studies on the basis of which we currently treat osteoporosis diverged from the STROBE criteria and employed statistical analyses that did not include correction for multiple comparisons [18–20].

Practical approaches: To mitigate the downsides, researchers often plan studies to limit the number of primary comparisons or use adaptive designs. Methods like hierarchical testing (sequentially testing outcomes until one fails) and FDR control offer more nuanced ways to handle multiplicity. Still, in many published studies (especially older or more conservative ones), Bonferroni remains the default adjustment. Understanding its effect on reported outcomes is crucial for anyone interpreting clinical research.

## Simulation study: evaluating P-value adjustments in multiple hypothesis testing

In this short simulation study, we aimed to evaluate the role of p-value adjustment in Type I and II errors in the context of multiple hypothesis testing.

We assume that we have two random samples, each of size * k=10*, drawn from two normal distributions: $\:N\left({\mu\:}_{0},{\sigma\:}^{2}\right)$ and $\:N\left({\mu\:}_{0}+\delta\:,{\sigma\:}^{2}\right)$, with $\:{\mu\:}_{0}=0$ and $\:\sigma\:=1$.

The objective was to test the hypothesis $\:{H}_{0}:\delta\:=0$ versus $\:{H}_{1}:\delta\:\ne\:0$.

To emulate the multiple comparisons scenario, we generated $\:n\:$independent hypothesis tests per iteration, with $\:n=\left\{1,\:5,\:10,\:30,\:50,\:100\right\}$.

The significance level was fixed at alpha = 0.05 (Type I error) and for each test, a two-sample *t*-test assuming equal but unknown variances was performed to assess equality of means between the two groups. The resulting *p*-values were then adjusted using five different methods:

(a) “None”: unadjusted *p*-values.

(b) “Bonferroni”: classical Bonferroni correction.

(c) “Holm”: sequential Holm-Bonferroni procedure [21].

(d) “FDR”: false discovery rate control via the Benjamini-Hochberg method [16, 22].

(e) “Bayes”: The empirical Bayes method of Stephens performs an adaptive shrinkage via the use of a unimodal prior resulting shrinkage that is adaptive to both the amount of signal in the data and the measurement precision of each observation [23].

**Fig. 2:**
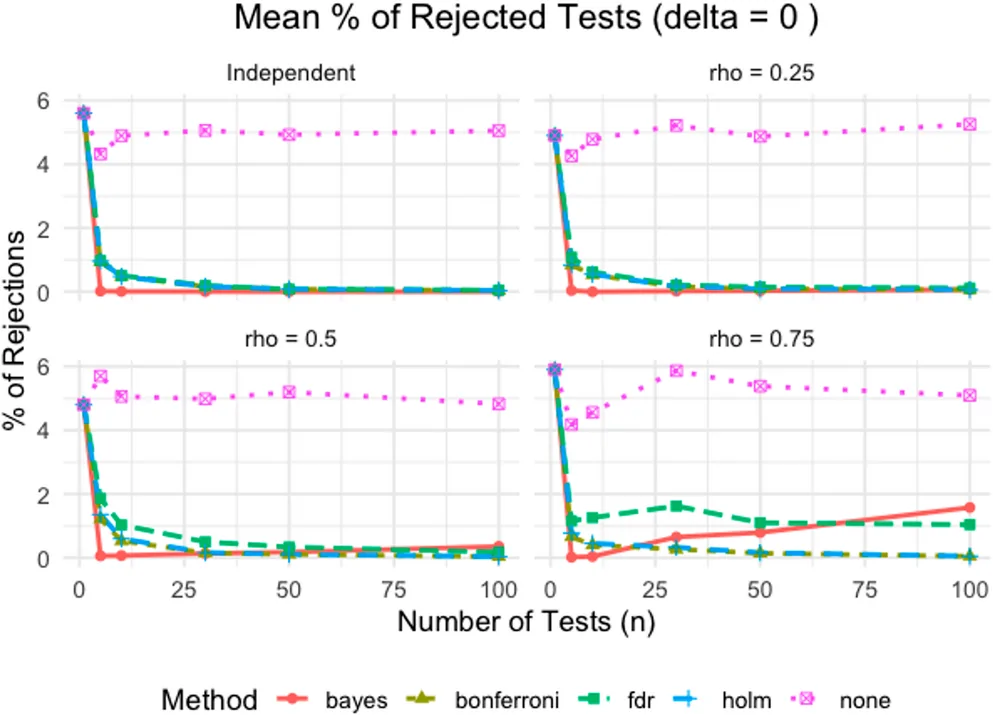
The Type I error versus the number of tests performed for the five competing methods, when we have independent (top left) or dependent samples

In each case, we recorded and plotted the percentage of tests for which H_0_ was rejected. For δ we used δ ∈ {0, 1, 2, 3}, where δ = 0 reflects Type I error performance (false positives), while δ ≠ 0 values investigate power for detecting effects of varying sizes. In addition, the two populations were either independent (ρ = 0) or dependent with ρ ∈ {0.25, 0.50, 0.75}. We ran the above methods 1,000 times for each configuration and estimated the mean (over iterations) percentage of tests that were rejected. When δ = 0 this refers to Type I error (false positives); smaller values are better. Figure 2 depicts the performance of the five competing methods.

Judging from Fig. 2, the Empirical Bayesian approach appears to be the best as long as the tests are not dependent. The Bonferroni and Holm corrections are nearly identical and outperform the rest when we have high dependence between tests yielding an extremely low percentage of false positives. The unadjusted p-values are at the level of alpha (0.05), while the FDR has a performance that is very close to the Bonferroni and Holm corrections, as long as we do not encounter high correlation among the tests, where it behaves similarly to the empirical Bayes adjustment.

**Fig. 3:**
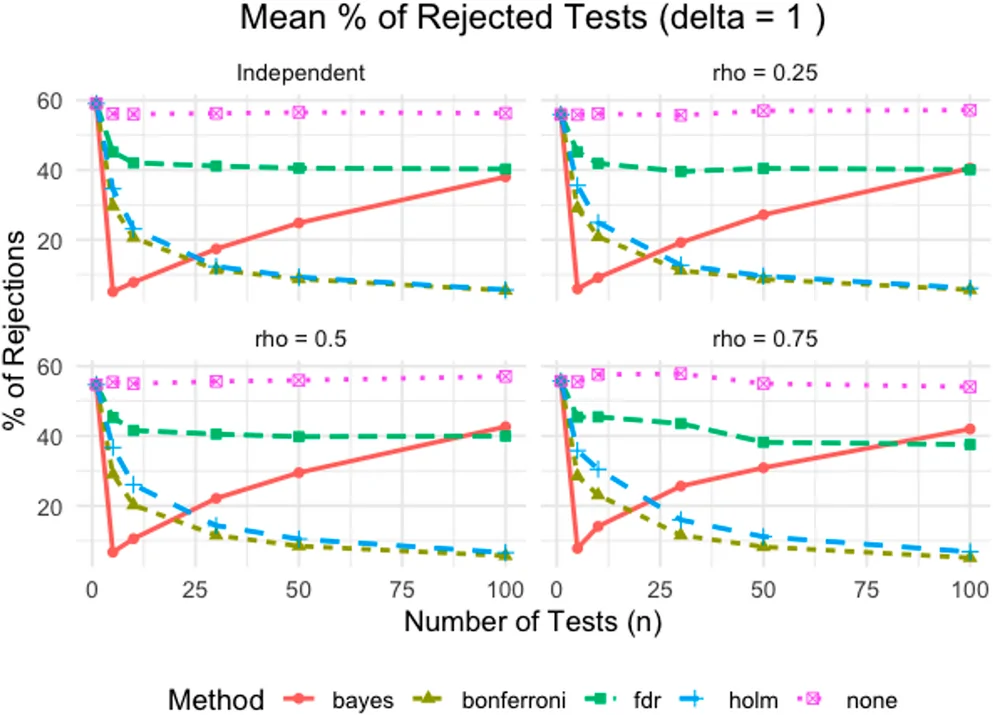
Detection power when $\:\delta\:=1$ versus the number of tests performed for the five competing methods, when we have independent (top left) or dependent samples

**Fig. 4:**
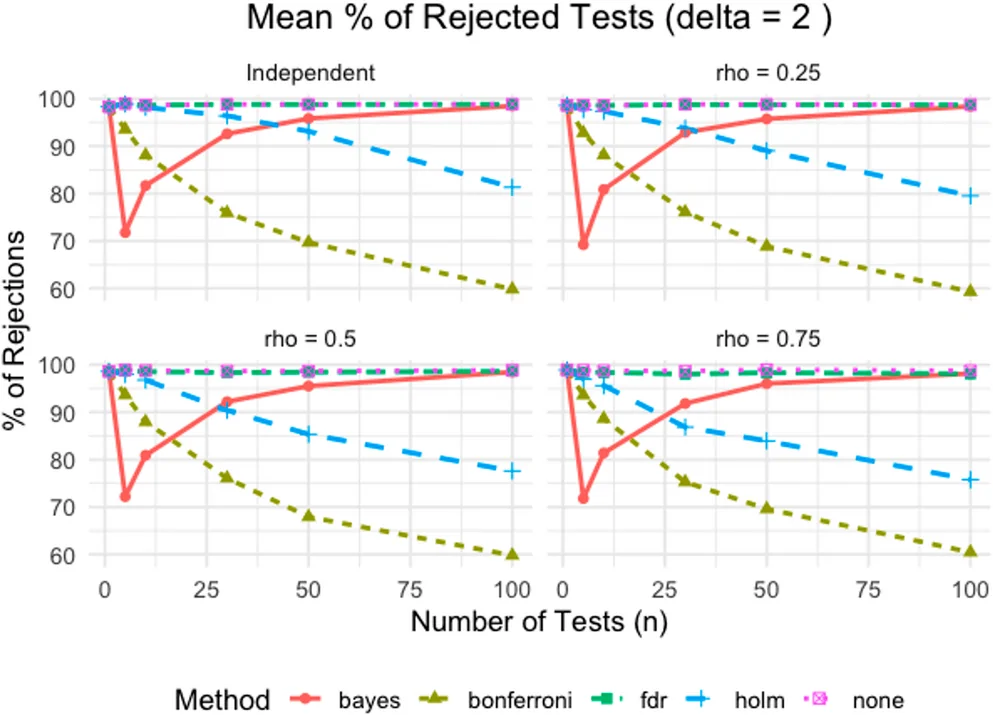
Detection power when $\:\delta\:=2$ versus the number of tests performed for the five competing methods, when we have independent (top left) or dependent samples

**Fig. 5:**
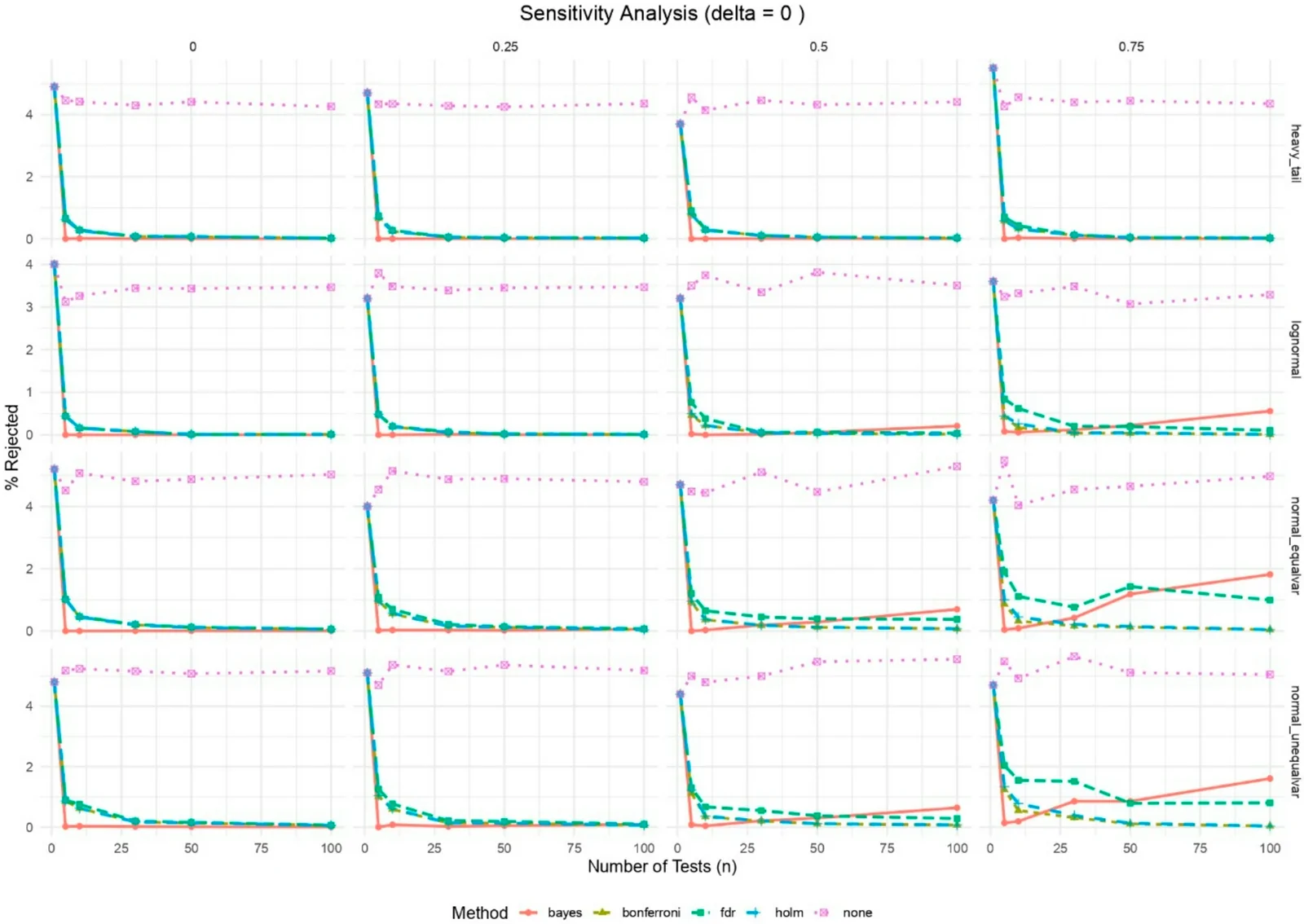
Detection power when $\:\delta\:=3$ versus the number of tests performed for the five competing methods, when we have independent (top left) or dependent samples

Regarding detection power, we consider that all * n* tests experience a mean shift of size $\:\delta\:\ne\:0$. The detection power (the higher the better) is reported in Figs. 3, 4 and 5 for the three sizes of non-zero $\:\delta\:$, respectively.

According to Figs. 3, 4 and 5, the Bonferroni correction exhibits the poorest performance, with an increasingly pronounced loss of detection power as the number of tests increases. For instance, in Fig. 4, when δ = 2 (a moderate-to-large effect size) and *n* = 50, approximately 30% of true effects are missed by the Bonferroni correction.The Holm correction is similar when we have small mean shifts, while it becomes increasingly better as the shift size increases. The empirical Bayesian approach works quite well, except in cases where we have a small number of simultaneous tests. Notably, apart from the small values of $\:\delta\:$, the FDR approach displays a performance that is almost identical to that of the unadjusted case.

**Fig. 6:**
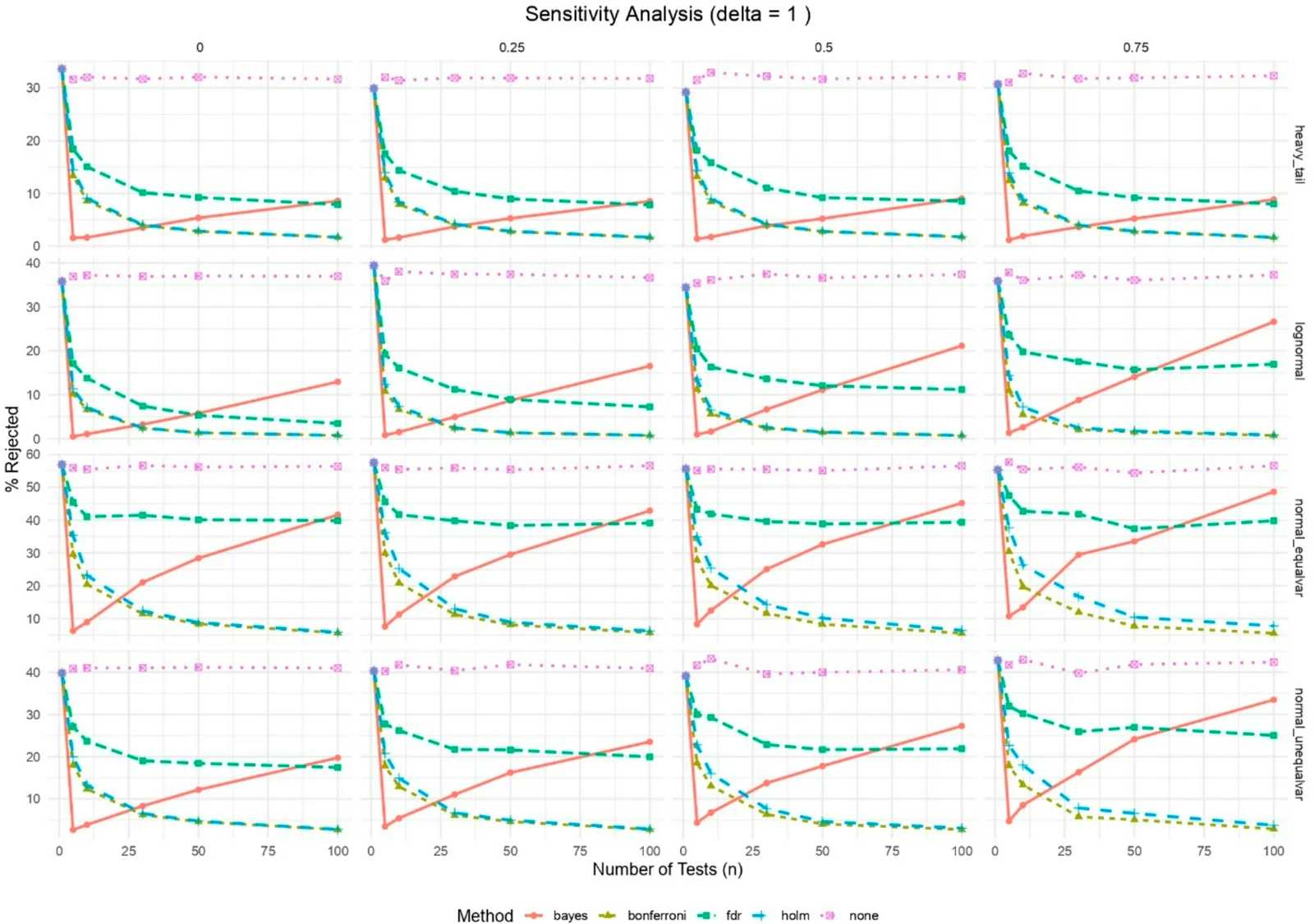
The FAR (top row) and TPR (bottom row), when $\:\delta\:=1$ versus the number of tests performed for the five competing methods, when we have independent or dependent samples

**Fig. 7:**
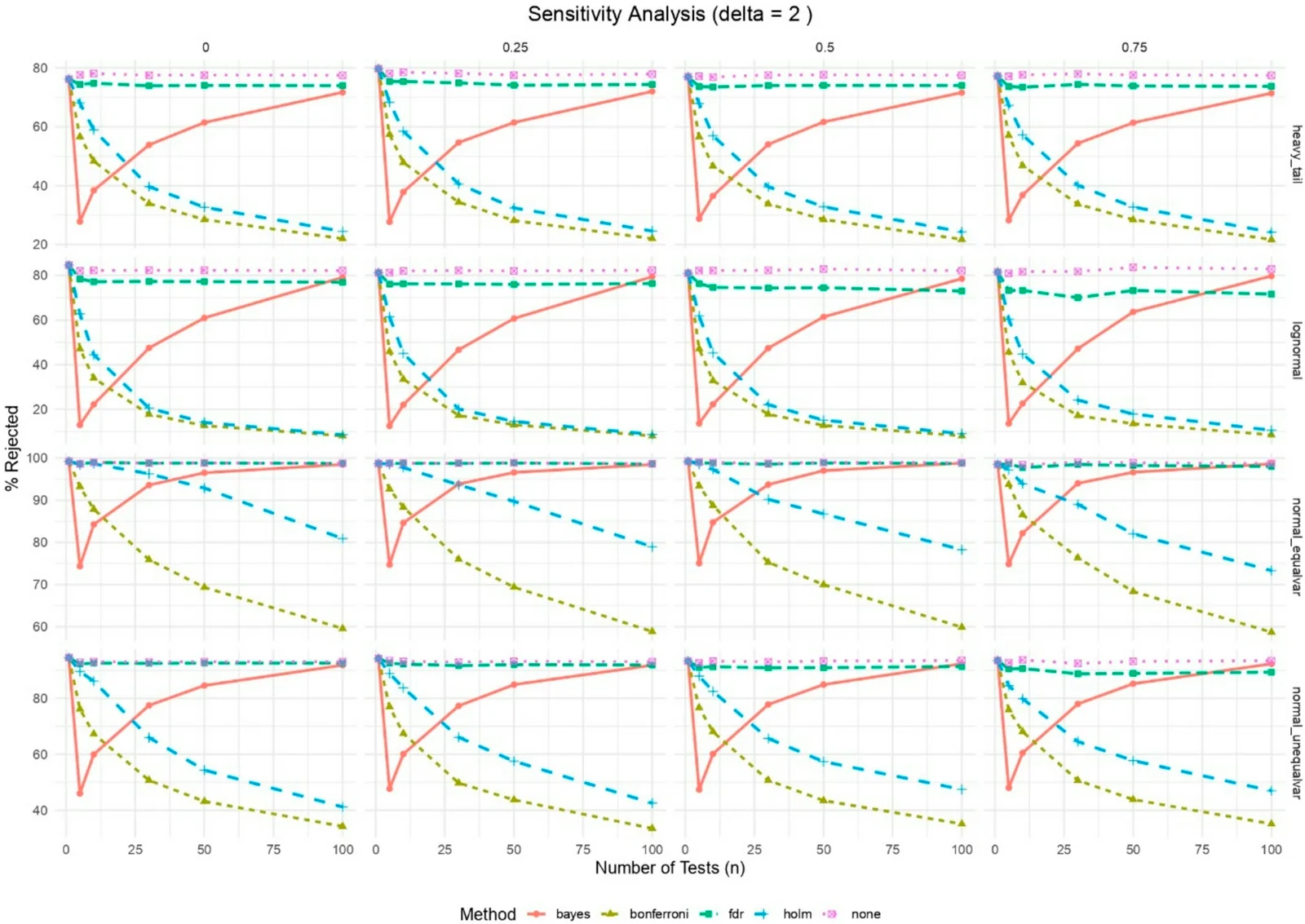
The FAR (top row) and TPR (bottom row) when $\:\delta\:=2$ versus the number of tests performed for the five competing methods, when we have independent or dependent samples

**Fig. 8:**
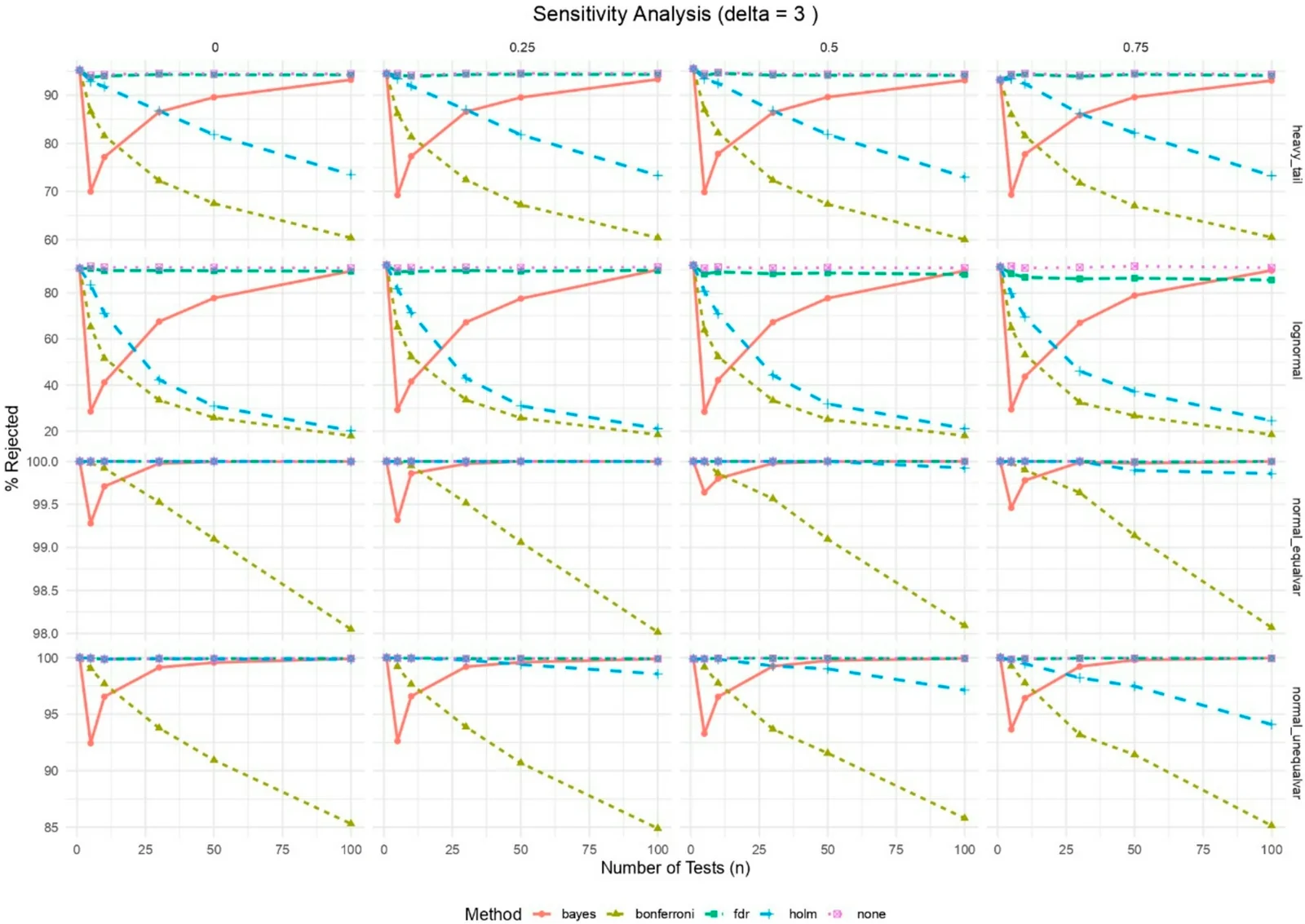
The FAR (top row) and TPR (bottom row), when $\:\delta\:=3$ versus the number of tests performed for the five competing methods, when we have independent or dependent samples

Finally, we have included another scenario where, among the * n* multiple tests performed, the effect of $\:\delta\:\ne\:0$ does not occur in all * n* simultaneous tests but only in 20% of them, while in the remaining 80% we have $\:\delta\:=0$. In Figs. 6, 7, and 8 we provide the false alarm rate (FAR), reflecting the Type I error (i.e., % of false positives in 80% of the tests with $\:\delta\:=0$) and the True Positive Rate (TPR), reflecting what percentage of the true effects were recognized (i.e. % of True Positives in 20% of the tests that had $\:\delta\:\ne\:0$).

In Figs. 6, 7 and 8 the relative performance of the methods is analogous to that in Figs. 3, 4 and 5: Bonferroni is extremely conservative, reducing detection performance, while FDR achieves a better balance between Type I and Type II errors. To provide numerical context to the qualitative trends shown in Figs. 2, 3, 4, 5, 6, 7 and 8; Table 2 summarizes the mean Type I error and detection power when δ = 2, *n* = 50 and all tests experience a mean shift.

**Table 2:** Mean type I error and detection power across multiple testing methods. The table quantifies numerically what we observe in the respective figures when δ = 2 for *n* = 50 and all tests experienced a mean shift. Specifically, we report the type I error and the power for all methods

Rho	Method	Mean Type I error	Mean detection power for δ = 2
0	bayes	2.00E-05	0.95842
0	bonferroni	0.0008	0.69698
0	fdr	0.0008	0.98802
0	holm	0.0008	0.93134
0	none	0.04926	0.98814
0.25	bayes	0.0003	0.95752
0.25	bonferroni	0.0009	0.68878
0.25	fdr	0.00148	0.9875
0.25	holm	0.00092	0.89044
0.25	none	0.0487	0.98806
0.5	bayes	0.00178	0.9552
0.5	bonferroni	0.0011	0.6793
0.5	fdr	0.00336	0.98462
0.5	holm	0.00112	0.85354
0.5	none	0.05196	0.98678
0.75	bayes	0.00792	0.9603
0.75	bonferroni	0.00142	0.69604
0.75	fdr	0.011	0.98328
0.75	holm	0.0016	0.83948
0.75	none	0.05382	0.98974

Please note that in this simulation study, we explicitly assume to have independent and identically distributed (iid) Normal data of size * k=10*. Violating these assumptions due to non-normality or autocorrelation or when bigger sample sizes are employed would yield potentially different results.

The simulation study was performed using the freeware R, version 4.2.2.

### Discussion of the simulation study

We performed a sensitivity analysis on the basic assumptions of normality and constant variance among the groups. Specifically, regarding normality, we considered two distributions, namely, lognormal and Student’s with 3 degrees of freedom, to examine the effect of either having right skewness or heavy tails, respectively. For the unequal variance scenario, we considered the case where the second group had double the variance of the first group. The findings are reported in extra sensitivity analysis figures where, in each case, the third row of plots refers to the valid assumption case (normal_equalvar) and the other three to the violation of normality (heavy_tail and lognormal) and violation of equality of variances (normal_unequalvar). Based on this study, we observe that the detection power is affected by these violations and all methods show decreased performance. However, the Empirical Bayes and the FDR methods appear to be less affected (more robust), providing the best overall performance, even under assumption violations.

## Conclusions

In conclusion, in real-life research settings, situations arise where strict adherence to STROBE guidelines—regarding randomization, the use of placebo arms, and/or corrections for multiple comparisons—may not be feasible or appropriate. In these instances, it is essential to devise alternative, rational routes of design and analysis that balance methodological rigor with practical and ethical considerations.

The Bonferroni correction serves as a potent example of this need for balance. While it acts as a double-edged sword in clinical studies—safeguarding against false positives and upholding a high standard for evidence—it carries the potential cost of overlooking meaningful findings that fail to surpass an arbitrarily strict threshold. Clinicians and researchers must remain mindful of this context. When Bonferroni adjustments significantly alter outcomes, it underscores that statistical significance is, in part, a function of analytical choices. A result fading from significant to non-significant after correction does not necessarily indicate the absence of an effect; rather, it reflects a cautious lens being applied.

Ultimately, striking the right balance in applying these and other methodological “corrections” is key to interpreting clinical evidence effectively and making informed decisions that weigh both the risks of false positives and the potential for missing true effects. By acknowledging the limitations of strict STROBE adherence in certain contexts and embracing a more nuanced approach to study design and statistical analysis, researchers can strive for the mesotes – the golden mean – that best reflects the truth and ultimately advances medical knowledge.

Calling urgent attention to the ongoing need for critical evaluation

When based on a solid foundation of high quality and replicable data, a consensus or guidelines statement among scholars who study a field can be useful; however, it should never become dogmatic. It is “scientific” to constantly question the value of and, indeed, intent of all consensus statements and guidelines. Their main objective is to reduce doubt, enabling public policy and general medical practice to move forward fast and with relative safety without further debate. Nonetheless, questioning and debate are central to science, and consensus statements and guidelines should not stifle scientific inquiry. Despite the obvious potential professional and social costs of questioning a consensus statement or guidelines emanating from concurring experts, researchers should always keep in mind that advances in knowledge have frequently upended consensus statements and guidelines. In the spirit of the Aristotelian mesotes, a balanced approach that embraces both guidance and critical evaluation will ultimately best serve the pursuit of the ideal, the most truthful approximation to reality.
